# Molecular and Immune Mechanisms Governing Cancer Metastasis, Including Dormancy, Microenvironmental Niches, and Tumor-Specific Programs

**DOI:** 10.3390/ijms27020875

**Published:** 2026-01-15

**Authors:** Dae Joong Kim

**Affiliations:** 1 Department of Orthopaedics, West Virginia University, Morgantown, WV 26506, USA; daejoong.kim@hsc.wvu.edu; 2 WVU Cancer Institute, Robert C. Byrd Health Sciences Center, West Virginia University, Morgantown, WV 26506, USA

**Keywords:** metastasis, disseminated tumor cells (DTCs), tumor dormancy, extracellular matrix (ECM), DNA methylation, osteosarcoma, chondrosarcoma, melanoma, hepatocellular carcinoma, breast cancer

## Abstract

Metastasis is still the leading cause of cancer-related death. It happens when disseminated tumor cells (DTCs) successfully navigate a series of steps and adapt to the unique conditions of distant organs. In this review, key molecular and immune mechanisms that shape metastatic spread, long-term survival, and eventual outgrowth are examined, with a focus on how tumor-intrinsic programs interact with extracellular matrix (ECM) remodeling, angiogenesis, and immune regulation. Gene networks that sustain tumor-cell plasticity and invasion are described, including EMT-linked transcription factors such as SNAIL and TWIST, as well as broader transcriptional regulators like SP1. Also, how epigenetic mechanisms, such as EZH2 activity, DNA methylation, chromatin remodeling, and noncoding RNAs, lock in pro-metastatic states and support adaptation under therapeutic pressure. Finally, proteases and matrix-modifying enzymes that physically and biochemically reshape tissues, including MMPs, uPA, cathepsins, LOX/LOXL2, and heparinase, are discussed for their roles in releasing stored growth signals and building permissive niches that enable seeding and colonization. In parallel, immune-evasion strategies that protect circulating and newly seeded tumor cells are discussed, including platelet-mediated shielding, suppressive myeloid populations, checkpoint signaling, and stromal barriers that exclude effector lymphocytes. A major focus is metastatic dormancy, cellular, angiogenic, and immune-mediated, framed as a reversible survival state regulated by stress signaling, adhesion cues, metabolic rewiring, and niche constraints, and as a key determinant of late relapse. Tumor-specific metastatic programs across mesenchymal malignancies (osteosarcoma, chondrosarcoma, and liposarcoma) and selected high-burden cancers (melanoma, hepatocellular carcinoma, glioblastoma, and breast cancer) are highlighted, emphasizing shared principles and divergent organotropisms. Emerging therapeutic strategies that target both the “seed” and the “soil” are also discussed, including immunotherapy combinations, stromal/ECM normalization, chemokine-axis inhibition, epigenetic reprogramming, and liquid-biopsy-enabled minimal residual disease monitoring, to prevent reactivation and improve durable control of metastatic disease.

## 1. Introduction

Metastasis, the process by which solid tumor cells migrate from the primary tumor to distant organs, is responsible for the majority of cancer-related deaths and exemplifies the complex interplay between tumor cells and their host microenvironments [1]. Although surgical procedures and radiotherapy can successfully eliminate the primary tumor, their therapeutic efficacy diminishes once tumor cells have spread throughout the body [2]. Disseminated tumor cells (DTCs) navigate through the bloodstream or lymphatic system, forming secondary lesions in distant organs. Metastatic dissemination depends on coordinated interactions between tumor cells and heterogeneous tumor-associated cells in the tumor microenvironment (TME) [3]. The diverse responses triggered by heterogeneous tumor cell treatment highlight the resistance characteristics associated with metastatic disease. Contemporary sequencing technologies have transformed our understanding of metastasis. Genetic studies of primary tumors, circulating tumor DNA, and circulating tumor cells have revealed significant differences and evolutionary changes between primary and metastatic tumors [4]. These methods allow real-time monitoring of disease progression, help identify biomarkers and treatment targets, and clarify how cancer cells use the body’s tissues to survive and grow [5].

Dormancy is a long-standing way for organisms to survive stress, and cancer cells seem to use this evolutionary program to wait out bad conditions [6]. When T cells enter a resting memory state, similar pathways are activated in immune cells. Tumor dormancy appears to hijack these conserved programs, allowing malignant cells to endure adverse conditions, such as immune pressure and toxic treatments, by entering a reversible growth arrest [7]. Dormancy may manifest locally before the proliferation of the primary tumor or systemically before the development of evident metastasis. Growing evidence indicates that metastatic dormancy may transpire before the identification of original tumors. These cells may disseminate throughout the body and remain quiescent in distant sites [6]. Their discovery is hindered by clinical limitations, particularly for lesions smaller than 5 mm [6]. Dormant cells can be single cells, small groups of cells, or tiny lesions that remain the same size due to balanced cell division and death [8]. The cancer cell’s own survival pathways, such as autophagy and stress response systems, as well as environmental factors, including the composition of the tissue scaffolding, tissue stiffness, and the availability of growth factors, control dormancy [8,9].

Metastasis is now recognized as a complex systemic process that requires collaboration between tumor cells and host tissues, encompassing tissue remodeling, immune evasion, angiogenesis, metabolic adaptation, and intercellular cooperation among various tumor cell types [2]. Finding hidden metastases early is a big problem when it comes to treating cancer. Evidence suggests that dissemination may transpire unexpectedly early in tumor development, potentially preceding clinical diagnosis [10]. Novel blood tests, such as the liquid biopsy platform, which detect circulating tumor cells, tumor DNA, or RNA-containing vesicles, have the potential to identify minimal disease in real time [11,12] and elucidate the formation and evolution of early metastatic colonies, integrating single-cell genetic analysis and spatial mapping techniques [5]. When imaging reveals metastases, they frequently exhibit distinct genetic profiles compared to the primary tumor due to pressures exerted by specific organs, variations in local tissue, and alterations occurring during treatment [3]. As a result, substantial heterogeneity is observed among metastases and even within individual metastatic foci, undermining the utility of conventional laboratory models and complicating the accurate prediction of treatment responses [13,14]. Current approaches to managing metastatic relapse are predicated on biomarkers derived from personalized strategies, reflecting the evolving biology of DTCs and their associated microenvironment [12]. Eliminating dormant cancer cells and preventing their reactivation are crucial for improving treatment outcomes in metastatic disease [8]. Immunological approaches, including modifying tumor-associated immune populations, boosting natural killer cell activity, and increasing tumor immunogenicity, offer potentially valuable strategies for targeting dormant and early DTCs [2,15]. Advancements in this domain depend on identifying dormancy-specific biomarkers that could enable early detection, guide risk-adjusted preventive measures, and ultimately reduce the incidence of metastatic recurrence [8]. While metastasis is a common focus of research in epithelial cancers, sarcomas, and other mesenchymal tumors, these tumors exhibit distinct metastatic behaviors and pose particular challenges. Unlike many carcinomas, which spread via lymphatic routes, sarcomas typically disseminate via the bloodstream, with a marked tendency to form lung metastases [16]. Furthermore, given their inherent mesenchymal characteristics, sarcomas might not depend on the conventional epithelial–mesenchymal transition (EMT) for their spread; indeed, certain sarcomas appear to undergo the inverse process, known as mesenchymal–epithelial transition (MET), during metastatic colonization [17,18]. This review will also cover how tumors reactivate and spread, focusing on the distinct biological features of mesenchymal malignancies, including osteosarcoma (OS), chondrosarcoma (CS), and liposarcoma (LPS).

## 2. Metastasis-Inducing Genes and Mechanisms

One of the hardest things about cancer is that it can spread to other parts of the body. It happens when cancer cells can leave where they started, travel through the bloodstream or lymphatic route, and settle in new places. Cells must change their shape and behavior, survive harsh conditions, and adapt to very harsh environments throughout the body. A deeper understanding of these mechanisms is essential for developing effective strategies to prevent cancer dissemination. Snail transcription factors are a group of proteins that help cancer cells make these changes (Table 1). *Snail1*, *Snail2* (also called *Slug*), and *Snail3* are the three types. They all help initiate a process that converts epithelial cells (which usually adhere in organized tissues) into mesenchymal cells (which can move more freely). Each one does something a little different. This change is an important step that allows cancer cells to acquire migratory and invasive capabilities.

A. SNAIL1: *Snail1*, the master orchestrator of the EMT, is the leading player in starting EMT. It works by turning off genes that keep epithelial cells together, such as *E-cadherin*, and altering the cells’ structure so they can move and invade more easily [30]. *Snail1* does this by binding to specific DNA sequences in the *E-cadherin* gene, preventing its transcription. Studies in mice show that Snail1 is essential at many stages of metastasis [31]. When cancer cells express active *Snail1*, they are much better at entering blood vessels and forming new tumors in the lungs. But *Snail1* does not just change the cancer cells. It also alters the surrounding tissue by turning normal support cells (fibroblasts) into cancer-supporting fibroblasts (cancer-associated fibroblasts, or CAFs). These cells have a very pro-invasive and inflammatory phenotype that helps the tumor grow and spread. These changed fibroblasts make prostaglandin E2 and enzymes that break down the scaffolding between cells. This makes it easier for cancer to move through tissues. This makes the environment stiffer, activating signaling pathways in tumor cells and helping them evade the immune system [32]. *Snail1* controls the spread of cancer by directly altering tumor cells and their environment.

B. SNAIL2 (SLUG): Many of its characteristics are very similar to those of *Snail1*. *Snail2* also inhibits adhesion molecules in epithelial cells, such as *E-cadherin*, which are involved in the initiation of the EMT program and facilitate cell movement. Although there is some overlap, *Snail2* demonstrates properties that distinguish it from the other Snail proteins [33]. A critical distinction is that it strongly influences the properties of cancer stem cells and their susceptibility to treatments. In the case of breast cancer, the high levels of *Snail2* are strictly associated with recurrence and metastasis, underscoring the role in the establishment of stem-like cells that can persist for a prolonged period and then give rise to metastasis [34]. *Snail2* is also involved in tumor metastasis to other parts of the body. This is because *Snail2* renders tumor cells resistant to the conditions to which they are subjected in the blood while circulating and enhances their adhesion to new sites. This is true in the cases of melanoma, colorectal carcinomas, glioblastoma (GBM), and hepatocellular carcinoma (HCC) [35]. Although *Snail2* can modulate the arrangement of the stromal cells, there is nothing very unique to this protein compared to *Snail1*. *Snail3* is a variant that is far more involved in the immune and developmental processes, while playing a minimal part in the overall development of metastasis [36].

C. The TWIST Family (Specialization in EMT and invasion).

The Twist family of basic helix-loop-helix (bHLH) transcription factors includes *Twist1* and *Twist2*. Both of these proteins are often inappropriately activated in cancer, and they play essential roles in the spread of cancer cells through distinct yet complementary mechanisms [37].

- *TWIST1* (Master EMT inducer and invasion coordinator)

*Twist1* is a key regulator of EMT. It stops *E-cadherin* and starts a wide range of transcriptional changes that make cells less sticky, change the structure of the cytoskeleton, and make cells move more easily [37]. *Twist1* not only plays a key role in EMT but also activates PDGFRα-Src signaling, which drives invadopodia formation.

These are actin-rich protrusions that bring together membrane-type 1 matrix metalloproteinase (MT1-MMP), MMP2, and MMP9 to create focused proteolysis at the leading edge. This way, *Twist1* helps tumor cells break through basement membranes and move into the stroma around them [20]. The increased expression of Twist1 in breast cancer and osteosarcoma (OS) is linked to more aggressive metastasis and worse clinical outcomes.

This shows that it is an important prognostic marker for metastasis across many tumor types [38].

- *TWIST2* (Improving survival and changing the immune system)

*Twist2*, while less well known than *Twist1*, plays essential roles in strengthening EMT programs and helping tumor cells survive. It boosts the levels of anti-apoptotic regulators like *Bcl-2* and *survivin*, which makes treatment less effective and leads to a disease that keeps coming back [39]. Twist2 also affects critical signaling pathways like *NF-κB* and *STAT3*, which help create conditions in the TME that suppress the immune system and make it harder for the immune system to find and kill the tumor [40].

D. Specificity Protein 1 (*SP1*): Master transcriptional coordinator specificity protein 1 (*SP1*) is a zinc-finger transcription factor with a wide range of pro-metastatic effects. It promotes EMT by activating major EMT regulators such as *Snail*, *Twist*, and *ZEB1*, and it increases the expression of matrix-modifying enzymes, including *MMP-2*, *MMP-9*, and *MT1-MMP*, that support extracellular matrix (ECM) degradation and tissue invasion (Table 1 and Table 2) [41]. 

In addition to EMT regulation, *Sp1* stimulates angiogenesis by directly inducing *VEGF* and *PDGF*, thereby supporting the vascular expansion required for tumor growth and metastatic spread [52]. *Sp1* enhances metastatic fitness by increasing the levels of survival factors such as *Bcl-2* and *survivin*, making cells more resistant to treatments that kill cancer cells [53]. Oncogenic signaling pathways, such as *EGFR*/*ERK* and *PI3K*/*AKT*, stabilize *Sp1* and create feedback loops that enhance its activity [54]. This enhances its transcriptional output. Increased *Sp1* expression is consistently associated with poor outcomes in multiple malignancies, such as breast, lung, colorectal, pancreatic, and liver cancers, highlighting its critical role in cancer progression [55].

## 3. Epigenetic Regulators of Metastasis

A wide variety of epigenetic regulators directly promote metastasis. *EZH2*, the catalytic subunit of PRC2, is one of the most important of these. *EZH2* represses tumor-suppressive genes, such as *E-cadherin*, by depositing H3K27me3. It also strengthens EMT by supporting transcription factors like *Snail* and *Slug* [56,57]. Inhibition of *EZH2* is associated with endothelial-mesenchymal transition (EndMT), whereas *EZH2* overexpression is significantly correlated with unfavorable prognosis across multiple carcinomas [58,59]. *EZH2*’s suppression of adhesion genes undermines epithelial integrity, thereby promoting invasion and triggering fibroblast-to-myofibroblast conversion, alongside wound-healing stromal remodeling, which in turn exacerbates metastasis [60]. Furthermore, a range of non-epigenetic oncogenic drivers contribute to metastatic progression. *PTTG1* (securin) promotes genomic instability and augments invasive behavior (Table 3) [61]. *BIRC5* (survivin) supports the survival of circulating tumor cells by inhibiting apoptosis [62]. *YBX1* regulates EMT, stemness, and treatment resistance [63]. Transcription factors such as *E2F1* and *MYB*, which govern the cell cycle, also activate gene programs associated with invasion [64,65]. These factors collectively facilitate invasion, survival, and microenvironmental remodeling throughout metastasis. Consequently, epigenetic reprogramming is essential for metastatic competence, as it confers the capacity to invade, disseminate, remain dormant, and establish secondary tumors.

DNA methylation: Abnormal methylation alters genes that control tumor growth. *DNMT1*, *DNMT3A*, and *DNMT3B* inhibit adhesion molecules like *CDH1* and enhance the expression of EMT transcription factors such as *Snail*, *Twist*, and *ZEB* [66]. Global hypomethylation at repetitive sequences and enhancers also activates oncogenic and inflammatory pathways that promote dissemination [67].

Histone methylation: EZH2-driven H3K27me3 inhibits tumor suppressors, lineage regulators, and immune-related genes [56,68]. Other methyltransferases, such as *G9a* (*EHMT2*; H3K9) and *SUV39H1*, contribute to the maintenance of mesenchymal states while simultaneously repressing the expression of epithelial identity [69]. The stability of epithelial-to-mesenchymal transition (EMT) programs and the facilitation of metastasis are further augmented by the absence of demethylases, including *KDM6A* (UTX) and *KDM6B* (JMJD3) [70].

**Table 3:** Dissemination, survival, and colonization.

Gene Name	Function	Description	Ref.
*CXCR4*	CXCL12 homing, organotropism	CXCR4 Chemokine receptor; guides cells to CXCL12-rich organs. Binds CXCL12 to direct cancer cell homing/migration to metastatic sites (lung, liver, bone). Endothelial cells produce CXCL12; CXCR4^+^ tumor cells adhere to the vasculature and extravasate. CXCR4 is expressed on fibroblasts/CAFs; CXCL12 from stroma promotes tumor-CAF interactions. Highly expressed on MSCs; mediates MSC homing and survival.	[71,72]
*FOXM1*	EMT, MMPs, angiogenesis	FOXM1 Forkhead TF; drives cell cycle, EMT (upregulates Snail/MMPs). In tumor cells, FOXM1 induces MMP2/9 and EMT factors, enhancing invasion. Promotes angiogenesis via VEGF expression; also implicated in EndMT in fibrosis. Shown to regulate CAF proliferation and extracellular proteases (in some tumors). May influence MSCs’ proliferative and migratory potential FOSL1 (FRA1) AP-1 subunit; EMT and invasion activator. Upregulates genes involved in motility (e.g., MMPs); promotes a mesenchymal phenotype. Stimulates VEGF and inflammatory cytokines, aiding vessel formation. In stromal cells, it supports the production of pro-tumorigenic ECM factors. In MSCs, differentiation may tilt toward a CAF-like state.	[73,74]
*E2F1*	Cell cycle invasion/angiogenesis programs	E2F1 Cell-cycle TF; pro-metastatic when overexpressed. Aside from proliferation, E2F1 can induce MMPs and EMT-associated genes. Drives expression of angiogenic factors (FGF, VEGF); can act in the endothelium. Linked to fibroblast proliferation; may contribute to desmoplasia. Activates proliferation of MSCs and endothelial precursors.	[75]
*MYB*	Stemness/invasion; angiogenic transcription	MYB Transcription factor can promote stemness and invasion. Activates target genes (including MMPs, EMT factors) in carcinomas. Regulates angiogenic gene expression (e.g., VEGFR). Influences fibroblast proliferation; MYB is expressed in some CAF subsets. Helps maintain MSC self-renewal; influences differentiation pathways.	[76]
*PTTG1* (Securin)	Genomic instability, EMT, invasion	PTTG1 (Securin) Promotes genetic instability and EMT. Overexpressed PTTG1 drives EMT and cell motility in cancer cells. May enhance secretion of angiogenic factors (through p53 inhibition). In fibroblasts, PTTG1 can promote proliferation and matrix production. In MSCs, PTTG1 supports proliferation, possibly aiding their tumorigenic roles.	[77,78]
*BIRC5* (Survivin)	Anoikis resistance, survival of CTCs/endothelium	BIRC5 (Survivin) Inhibitor of apoptosis; cell division regulator. Upregulated in metastatic tumors to allow anoikis resistance and survival in circulation. Supports the survival of proliferating endothelium in tumor vessels. Protects CAFs/myofibroblasts from apoptosis, sustaining pro-metastatic stroma. Ensures MSC survival in harsh metastatic niches.	[79,80]

Histone acetylation and deacetylation: Histone acetyltransferases (*p300*/*CBP*) activate EMT drivers [81], and in the context of metastatic disease, they suppress epithelial programs while promoting angiogenesis and immune evasion [82]. Consequently, HDAC inhibitors have the potential to reverse EMT characteristics and improve responses to immunotherapy [83].

Chromatin remodeling: SWI/SNF (BAF) complexes impact metastasis, although the effects are context-dependent. The absence of *ARID1A* or *SMARCA4*, for instance, enhances dissemination across various cancers, whereas distinct BAF configurations can promote invasion and therapeutic resistance [84].

Noncoding RNAs and interactions: Long noncoding RNAs and microRNAs regulate chromatin-modifying enzymes. *HOTAIR*, for instance, brings PRC2 to shut down epithelial genes and speeds up EMT and distant metastasis in breast cancer [85]. The reversibility of epigenetic modifications presents therapeutic prospects in clinical settings. Targeting epigenetic plasticity thus constitutes a promising strategy to halt or treat metastatic progression. Human cancer studies have linked each of the genes above to cancer spread, usually through the mechanisms described. For example, *Snail1* and *Twist1* have been shown to drive invasion through EMT programs directly. Interleukin-6 (*IL-6*) and Interleukin-8 (*IL-8*) are well-known cytokines that promote metastatic phenotypes in many cell types, and MMPs degrade the ECM, enabling cells to spread.

## 4. Extracellular Proteases and Matrix Modifiers in Metastatic Progression

Recent accumulating evidence consistently identifies the ECM as a pivotal factor influencing cancer risk and progression. For instance, women with breast tissue that is rich in collagen have a fourfold higher risk of getting breast cancer [86]. In established tumors, the ECM serves as both a structural framework and a source of biochemical signals. Metastatic tumor cells release a variety of extracellular proteases and matrix-modifying enzymes, which instigate significant alterations to the ECM. This remodeling process destabilizes the matrix’s structure, liberates growth factors sequestered within the ECM, and produces bioactive matrix fragments. These alterations, in turn, facilitate invasion, angiogenesis, inflammation, and the establishment of pre-metastatic niches. Furthermore, cross-species xenograft investigations provide additional evidence that ECM remodeling results from the combined, yet separate, actions of tumor cells and stromal elements. Tumor cells primarily synthesize matrix-modifying and matrix-degrading enzymes, while stromal fibroblasts and other mesenchymal cells predominantly provide ECM glycoproteins; both cell types are involved in the production of fibrillar collagens [87]. *Collagen I*, specifically, is consistently elevated in primary tumors of the breast, liver, lung, and esophagus, and is similarly heightened in metastatic ovarian cancer [88,89,90]. Hyaluronic acid (HA) builds up in breast, prostate, bladder, and colon cancers and is linked to metastasis and a bad outcome [91,92]. Elevated epithelial fibronectin in breast cancer indicates diminished survival. *Laminin-111* is reduced in tumors compared to normal tissues [93], whereas *laminin-332* is elevated in various cancers [94], including breast cancer, and is associated with a poorer prognosis. In advanced omental metastases of ovarian cancer, there are more glycoproteins (like fibrinogen and fibronectin), proteoglycans, secreted factors, and ECM-associated proteins than in smaller lesions [95]. Proteomic analyses have identified *tenascin-C* as a prognostic marker in lung cancer and *SERPINB1* as a factor influencing brain tropism in breast cancer metastasis [96]. RGD-modified HA improves cooperative chemotherapy resistance in GBM [97], PEG hydrogels with MMP-degradable linkers enable GBM cells to develop actin-rich protrusions, while non-degradable matrices confine cells to a rounded morphology [97].

A. Functions of the matrix metalloproteinase family

MMPs are the most well-known extracellular proteases in cancer. *MMP-9*, a highly active gelatinase, is abundant in aggressive tumors. It releases growth factors like *VEGF* and *TGF-β* that are bound to the matrix, thereby strengthening signals that drive invasion and angiogenesis. *MMP-1*, an interstitial collagenase, cleaves fibrillar collagens, making it easier for stromal cells to enter tissues and penetrate them [98]. *MMP-9* facilitates various stages of metastatic progression, encompassing EMT induction, enhanced cell migration, angiogenic activation, and the sustenance of tumor-associated inflammatory responses [99]. *MMP-9* remodels collagen structure and promotes endothelial sprouting at invasive fronts. This makes neovascularization easier, which is necessary for tumor growth and spread [100]. *MMP-9* from tumors facilitates intravasation, while MMPs from CAFs alter the stroma, making it stiffer to help the tumor keep invading [101].

B. Additional protease systems

High uPA pathway activity is consistently associated with poorer prognosis in breast and colorectal cancers, underscoring its clinical significance [102]. This system performs necessary supplementary functions to MMP systems by degrading fibrillar collagens and components of the basement membrane. Cathepsins, particularly *cathepsins B*, *L*, and *S*, enhance matrix-degrading capabilities and are instrumental in compromising vascular barriers, thereby facilitating intravasation and aiding metastatic dissemination [103].

C. Enzymes that change the matrix and control biomechanics

Tumor cells release specialized matrix-modifying enzymes that alter the biomechanical properties of the ECM, thereby accelerating cancer spread. The lysyl oxidase (*LOX*) family of enzymes links collagen fibers together, which makes the matrix stiffer. This rigidity triggers mechanosensitive signaling pathways that include integrins, *FAK*, and *RhoA*, which help cells move. In breast cancer research, hypoxic conditions stimulate *LOX*, which remodels collagen and creates a pre-metastatic niche in the lung [104]. *LOXL2*, a related enzyme, stabilizes EMT transcription factors like *Snail* by chemically changing them after they are made. This strengthens mesenchymal traits and makes cells more likely to spread to other parts of the body [105]. Heparanase (*HPSE*) alters the ECM by cleaving heparan sulfate proteoglycans, which releases angiogenic and pro-invasive factors like *FGF* and *VEGF* and makes it easier for tumor cells to move around [106].

## 5. Mechanisms of Immune Evasion in Metastatic Cancer

Immune evasion during metastasis is facilitated by several interconnected mechanisms, encompassing suppressive immune cells, checkpoint signals, and physical impediments within the tissue microenvironment. Monocytic myeloid-derived suppressor cells (MDSCs) and tumor-associated macrophages (TAMs) undermine immune function by releasing immunosuppressive molecules, such as *TGF-β*, reactive oxygen species, and arginase, thereby curtailing the cytotoxic potential of T cells and natural killer (NK) cells. Tumor-associated macrophages (TAMs) frequently exhibit a phenotypic transition toward an alternatively activated M2-like state, thereby promoting angiogenesis and facilitating the tumor’s evasion of immune surveillance [107]. Moreover, platelets play a role in shielding tumor cells from the immune system by encapsulating them as they circulate through the bloodstream. Platelets also release thromboxane A2, which can suppress T-cell activation through the ARHGEF1 signaling pathway. Consequently, pharmacological inhibition of platelets substantially diminishes the metastatic potential to spread to distant organs, implying that this strategy may be beneficial in patient treatment [108]. In existing metastases, T-cell function is often weakened by various immune checkpoint pathways. Specifically, in metastases, especially in areas like the brain that are protected, T cells that have entered the tumor often show high levels of inhibitory receptors such as *PD-1*, *LAG-3*, and *TIGIT* [109]. In addition, the TME has strong stromal barriers that prevent immune cells from reaching cancer cells. A thick ECM and the presence of CAFs can physically prevent effector immune cells from reaching tumor sites. For instance, in pancreatic cancer, a dense stroma rich in hyaluronan impedes blood flow and prevents T cells from entering the tumor core. Research in the lab has shown that using enzymes to break down these matrix components can make it easier for drugs to enter cells and for T cells to enter the stroma. This is evidence that strategies to normalize the stroma could be helpful [110].

## 6. Cytokine and Chemokine Networks in Metastatic Dissemination

Cytokines and chemokines are the main factors that control how cancer cells spread throughout the body. They create communication networks that guide cancer cells, help create areas where metastasis can begin, and manage the complex interactions between cancer cells and the body’s tissues.

A. Chemokine-driven organ targeting

The CXCL12-CXCR4 pathway is the most well-studied chemokine pathway, and it plays a key role in how cancer cells migrate to different organs. It directs breast and prostate cancer cells to *CXCL12*-rich tissues, including the lung, liver, and bone marrow, where elevated chemokine concentrations establish conducive environments for incoming tumor cells [111]. The *CCR7-CCL21* chemokine axis facilitates lymphatic dissemination, as *CCL21* is continuously expressed by lymphatic endothelial cells and lymph node stromal cells, directing *CCR7*-expressing tumor cells towards lymphatic vessels and draining lymph nodes [112]. The *CCR9-CCL25* axis also causes metastasis in the gut by directing tumor cells to intestinal sites that are rich in *CCL25* [113].

B. Pro-metastatic cytokine signaling

Cytokine networks orchestrate metastasis through a variety of interconnected mechanisms. For instance, *IL-6* initiates *JAK*/*STAT3* signaling, thereby fostering epithelial–mesenchymal transition (EMT) and the acquisition of cancer stem cell characteristics; this was observed in gastric cancer studies investigating the interplay between CAFs and tumor cells [114]. The effects of *TGF-β* are context-dependent. Initially, it acts as a tumor suppressor, yet in later stages of cancer, it promotes EMT, immune evasion, and metastasis [115,116]. Furthermore, *TNF-α* augments invasion by activating *NF-κB*, which subsequently increases the expression of adhesion molecules and matrix metalloproteinases, thereby facilitating cellular infiltration and tissue remodeling.

Coordinated signaling between *IL-1β* and *CXCL8*/*IL-8* promotes neutrophil recruitment, stimulates tumor-angiogenesis, and provokes EMT programs at invasive tumor margins, which facilitate inflammatory microenvironments that drive tumor progression [117].

C. Cytokine networks in the pre-metastatic niche

Certain cytokine combinations, such as *VEGF-A*, *TNF-α*, and *TGF-β*, act together to increase levels of *S100A8*/*A9* and fibronectin (*FN1*) in the lung’s supporting tissue during the formation of pre-metastatic niches. This methodically brings in myeloid cells to set up conditions that make it easier for tumor cells to settle later [118]. In patients, elevated levels of chemokines and cytokines are significantly associated with adverse outcomes, preferences for particular metastatic sites, and treatment resistance. Because of these results, clinical trials are now testing targeted therapies like *CXCR4* inhibitors (plerixafor), *CCR5* inhibitors (maraviroc, NCT01736813), *IL-6* receptor antagonists (tocilizumab, NCT03999749), and *TGF-β* antagonists for the treatment of metastatic disease [119].

## 7. Pre-Metastatic Niche Formation and Organ-Specific Colonization

A. Niche preparation orchestrated by the primary tumor

Primary tumors send signals that prepare other organs for cancer cells to move in long before they actually do. Significant research has shown that bone marrow cells expressing *FLT1* (VEGFR1) travel to these distant sites in response to molecules released by the original tumor. This creates a support structure that makes it easier for metastases to grow later [120]. *LOX* is essential for this preparation because it crosslinks collagen molecules and attracts myeloid cells that remodel tissue structure in target organs like the lungs. It is made when tumors have low oxygen levels. VEGFA and related chemokine signaling pathways promote the recruitment of immunosuppressive cell populations, establishing a microenvironment that is permissive for metastatic seeding and outgrowth [121].

B. Communication between cells through exosomes

Exosomes released by primary tumors serve as communication vehicles that instruct resident fibroblasts and endothelial cells in remote organs on their behavior. These small vesicles also alter the function of circulating neutrophils and monocytes, turning them into cells that either help build new blood vessels or suppress immune responses. This makes it easier for metastatic cells to settle in later [122].

C. Organ-specific niche architecture and upkeep

Pre-metastatic niches (PMNs) form in a way that is specific to the biology of each target organ. In the lung, early PMNs exhibit significant neutrophil clusters and infiltration by inflammatory macrophages. In bone, homing mediated by *CXCR4-CXCL12* and signals that activate osteoclasts are very important. The formation of PMNs in the brain depends on specific adhesion interactions with endothelial cells, along with changes in the blood–brain barrier. After these areas are prepared, resident stromal cells support the growth of metastases through organ-specific processes. For example, in the lungs, perivascular fibroblasts produce tenascin-C (*TNC*), which attracts and activates macrophages. This strengthens a microenvironment that helps cancer cells spread [123].

## 8. Tumor Dormancy

**Figure 1:**
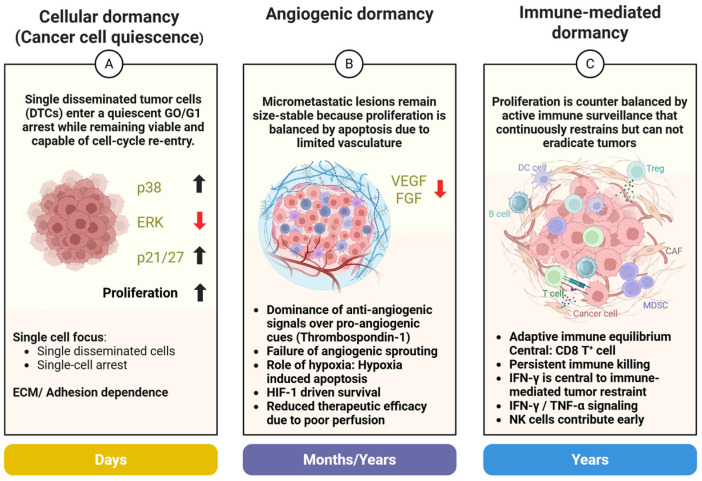
Distinct but overlapping mechanisms of tumor dormancy. Tumor dormancy encompasses biologically distinct states in which cancer cells or lesions persist without net expansion yet retain the capacity for reactivation. (**A**) Cellular dormancy represents a cell-intrinsic, reversible quiescent state in which single disseminated tumor cells (DTCs) undergo G0/G1 arrest while remaining viable and capable of cell-cycle re-entry. This state is characterized by a high p38-to-ERK signaling ratio, induction of cyclin-dependent kinase inhibitors (p21^Cip1^, p27^Kip1^), suppressed proliferation, and strong dependence on ECM and adhesion cues. (**B**) Angiogenic dormancy is a population-level constraint in which micrometastatic lesions remain size-stable because tumor cell proliferation is balanced by apoptosis due to insufficient vascularization. Dominance of anti-angiogenic signals over pro-angiogenic cues prevents completion of the angiogenic switch, resulting in hypoxia, *HIF-1*-dependent survival programs, impaired neovascularization, and reduced therapeutic efficacy owing to poor perfusion. (**C**) Immune-mediated dormancy reflects an adaptive immune equilibrium in which tumor cell proliferation is counterbalanced by continuous immune surveillance, preventing net tumor expansion without complete eradication. Tumor-reactive CD8^+^ T cells, supported by IFN-γ and TNF-α signaling, are central to maintaining immune restraint, with early contributions from innate immune cells. These dormancy mechanisms can coexist or occur sequentially and operate over overlapping temporal scales, ranging from days to years, ultimately shaping metastatic persistence and relapse.

Tumor dormancy refers to a state in which cancer cells or tumors stop expanding but remain alive and capable of regrowth. During dormancy, cancer progression is paused, either individual cells lie quietly without dividing (cellular dormancy), or a microscopic tumor mass remains size-stable due to balanced cell birth and death (tumor mass dormancy) [124,125]. Dormancy can persist for years, consistent with late relapse after apparently curative therapy [124,125]. Crucially, dormancy is reversible. Permissive cues can trigger dormant cells or lesions to “wake up” and resume growth [126]. Three major mechanisms are commonly distinguished: cellular dormancy, angiogenic dormancy, and immune-mediated dormancy (Figure 1), each with characteristic biology and therapeutic implications [126,127].

A. Cellular dormancy (Cancer cell quiescence)

Cellular dormancy is an intrinsic, single-cell growth arrest in which DTCs enter a reversible G0/G1 quiescent state and stop proliferating [124,125]. These cells remain viable yet non-dividing, often with reduced biosynthetic activity, enabling survival under stress (growth factor deprivation, ECM detachment, therapy, etc.) [128]. A canonical hallmark is a high p38 MAPK-to-ERK signaling ratio, where stress-activated p38 and suppressed ERK support dormancy via induction of cell-cycle inhibitors (e.g., p21^Cip1^, p27^Kip1^) and repression of proliferative programs [128,129]. Loss or alteration of adhesion/ECM signaling can enforce this dormant signaling state; integrin pathway perturbation (including β1-integrin-dependent programs) is linked to escape from dormancy and emergence of permissive microenvironments [130,131]. Dormant cells also engage stress-survival adaptations (e.g., redox control, metabolic rewiring) that preserve viability during nutrient/oxygen limitation [132,133,134]. Dormancy is frequently shaped by niche ECM composition; collagen-rich or specialized ECM niches can actively restrain outgrowth and stabilize dormant states [135].

B. Angiogenic dormancy

Angiogenic dormancy is a tumor-level dormancy caused by inadequate neovascularization. Here, micrometastases or microscopic tumors cannot expand because they fail to complete the angiogenic switch, yielding a net equilibrium where proliferation is offset by apoptosis due to hypoxia/nutrient limitation [127,132,136,137,138]. Key features and mechanisms: Angiogenic dormancy reflects a balance between pro-angiogenic mediators (e.g., VEGF) and endogenous anti-angiogenic signals (e.g., thrombospondin-1), keeping vascular expansion constrained [127,136,139]. In classic in vivo models, VEGF-dependent angiogenic switching is nonredundant for progression from microscopic lesions to expanding tumors [136]. Hypoxia/*HIF-1*-dependent adaptation can support survival and phenotypic evolution in poorly perfused tumors, contributing to persistence during vascular restraint and facilitating later escape when vascular constraints relax [132,140,141,142]. When the pro-angiogenic balance tips (e.g., increased VEGF activity or reduced anti-angiogenic restraint), lesions can rapidly vascularize and transition from dormancy to overt growth [127,136].

C. Immune-mediated dormancy

Immune-mediated dormancy occurs when immune surveillance maintains cancer in an equilibrium, tumor cells may persist and even divide, but cytotoxic elimination prevents net expansion, keeping disease clinically occult or stable [124,126]. Adaptive immunity, particularly tumor-reactive CD8^+^ T cells with support from Th1-polarized CD4^+^ T cells, can restrain outgrowth by continuous immune pressure and cytokine-mediated enforcement of non-progression [126,143]. *IFN-γ* and *TNF-α*-linked immune programs can constrain tumor expansion and promote non-progressive states under sustained immune surveillance [126]. In melanoma, tissue-resident memory CD8^+^ T cells have been implicated in maintaining long-term tumor-immune equilibrium consistent with immune-mediated restraint [143]. When immune pressure is removed or fails (e.g., T-cell dysfunction/exhaustion), escape and rapid outgrowth can occur [126]. Immune editing during this equilibrium may also select for less immunogenic variants, including antigen-presentation alterations, which can facilitate eventual relapse [124,126].

D. Comparing the three dormancy mechanisms

Cellular dormancy is fundamentally cell-intrinsic (single cells enter quiescence), whereas angiogenic and immune dormancy are microenvironment-constrained population states in which proliferation and death remain dynamically balanced [124,125]. Angiogenic dormancy is limited by vascular supply, while immune-mediated dormancy is limited by immune-mediated killing/containment [124,126,136]. These mechanisms can overlap or occur sequentially within the same patient, as DTCs transition between niche-imposed constraints and intrinsic quiescence programs [124,126,127,135]. Distinguishing these forms of dormancy is clinically actionable because each implies different intervention logic: maintain dormancy (anti-angiogenic therapy, immune reinforcement) versus eradicate dormant reservoirs by targeting quiescence-survival dependencies, niche ECM support, or immune-escape pathways [124,125,126,127].

## 9. Tumor Metastasis Mechanisms by Tumor Type

Metastasis results from a highly complex bidirectional interaction between cancer cells, the stromal microenvironment, and host immune system components, and it has remained the leading cause of cancer-related death. This section brings together tumor-specific metastatic phenomena with fundamental biological processes that regulate dissemination, long-term survival, and growth in a secondary organ setting. Special attention will be given to phenomena in which DTCs remain in a quiescent, indolent phase and subsequently undergo reactivation, marking fundamental transition events in a metastatic setting.

Among these mechanisms are cancer cell phenotypic plasticity, ECM remodeling, modulation of stromal and immune cells, and the creation of permissive niches for dormancy or metastasis. By considering metastasis as a process of dormancy and activation, there is scope to study underlying biological themes across different tumor systems and tumor-specific variations. Instead of going through a general discussion of each of the prominent types of malignancy of the epithelial lineage, this segment of the discussion will focus on malignancies in which dormancy mechanisms as well as those related to the reactivation of dormancy have not been as thoroughly synthesized from a biological perspective, or have divergent characteristics, like osteosarcoma, chondrosarcoma, liposarcoma, melanoma, hepatocellular carcinoma, GBM, and breast cancer. While malignancies like lung cancer, colon cancer, pancreatic cancer, and stomach cancer, among others, have a very high prevalence rate, none of these will be discussed with the same level of prominence. However, the fundamental mechanisms that promote dormancy, as well as those related to the re-entry of cancer into its active phase, share a high degree of overlap.

### 9.1. Osteosarcoma (OS)

**Figure 2:**
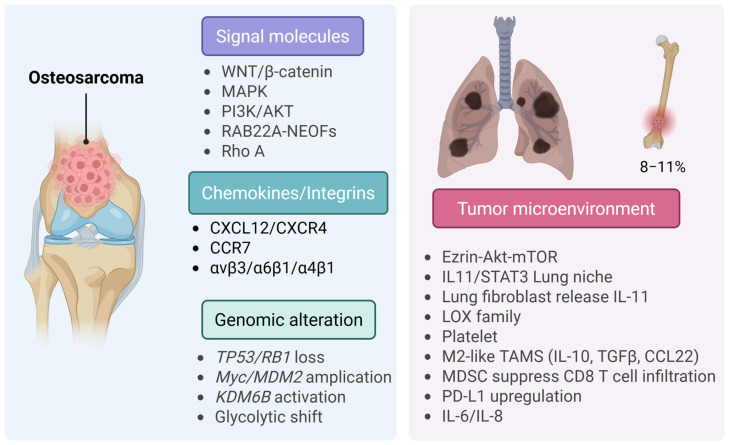
Schematic overview of tumor-intrinsic and microenvironmental drivers of osteosarcoma (OS) lung metastasis. The left panel depicts tumor-derived pro-metastatic mechanisms intrinsic to OS cells, including dysregulated signaling pathways, chemokine/integrin expression, and oncogenic genomic alterations that together endow cancer cells with heightened invasiveness, survival advantages, and the capacity to evade immune attack. The right panel illustrates the metastatic niche in the lung, which is conditioned by both tumor-secreted factors (e.g., growth factors, cytokines, extracellular vesicles) and host responses (such as immune cell recruitment and stromal activation), creating a permissive microenvironment for tumor engraftment. Together, these molecular and cellular interactions cooperate to promote OS cell invasion into lung tissue, resistance to immune clearance, and successful colonization and outgrowth of metastatic lesions in the lung.

Early metastasis, particularly to the lungs, is the principal factor influencing prognosis (Managing the immune microenvironment of OS and treatment). About 15–20% of patients have metastases at the time of diagnosis. Of those who are first diagnosed with localized disease, 30–40% will eventually have a relapse, most often with pulmonary disease. About 80–90% of distant metastases affect the lungs, with secondary bone involvement at approximately 8–11%, and, infrequently, visceral or CNS locations [138,144,145]. The lung is still the most common place for OS to come back and the most common cause of death from OS (The metastatic patterns of OS). At the genomic level, OS exhibits significant chromosomal instability and persistent disruption of established tumor-suppressor pathways (Figure 2) [146]. The most common type of loss-of-function in *TP53* and *RB1* is when *CDKN2A* is deleted, and *MYC*/*MDM2* is amplified [147]. These changes happen as part of complicated structural changes that help cancer cells adapt and spread. The *Rab22a-NeoF1* fusion facilitates lung colonization by activating *RhoA*, remodeling the cytoskeleton, and forming invadopodia. Importantly, exosomes containing *Rab22a-NeoF1* prepare pre-metastatic lung niches [148]. The histone demethylase *KDM6B* increases glycolytic flux by upregulating LDHA, which supports metastatic growth [149]. Similarly, the m6A RNA demethylases FTO and ALKBH5 control post-transcriptional processes that strengthen metastatic traits, such as increased cell invasiveness and improved stress resistance [150]. Metastatic OS cells frequently demonstrate significant aerobic glycolysis (the Warburg effect), which promotes anabolic metabolism, supports survival in hypoxic environments, and leads to the acidification of the surrounding microenvironment [151]. The TME is therefore a central determinant of OS progression and metastatic competence [152]. TAMs, especially M2-polarized subsets, are associated with a poor prognosis and increased lung colonization because they release pro-angiogenic and immunosuppressive factors. Matrix metalloproteinases, especially *MMP-9*, help the ECM change shape and release growth factors [153]. Platelets shield circulating tumor cells from shear stress and immune surveillance, promoting vascular adhesion and metastatic seeding [154]. The pulmonary niche signals that help cells survive, and lung-derived mediators activate MAPK signaling and increase the levels of anti-apoptotic molecules like *MCL1*, which supports micrometastatic growth [155]. Metastatic competence reflects the integration of various oncogenic pathways. *PI3K*/*AKT* improves survival and metabolic adaptation; MAPK/ERK promotes proliferation and invasion; WNT/β-catenin controls stemness and motility; and *Rho*/*Rac* GTPases manage cytoskeletal remodeling [156]. Chemokine networks, particularly the *CXCL12*/*CXCR4*/*CCR7* axis, play a crucial role in guiding tumor cells to their destinations. Integrins, including *αVβ3* and *αVβ5*, are essential for firm cell adhesion to the endothelium and the ECM. Consequently, pharmacological inhibition of these integrins has been shown to reduce metastasis to the lungs in preclinical models [157,158]. Furthermore, experimental orthotopic and intravenous models, which employ highly metastatic cell lines (e.g., K7M2, 143B) and genetically engineered murine models that display conditional *p53*/*Rb* loss in osteoblasts, have enabled a comprehensive investigation of the mechanisms that underlie lung metastasis [159,160]. Clinical genomic datasets validate these findings, underscoring the significance of *TP53*/*RB1* disruption and pulmonary relapse as primary contributors to treatment failure [147]. The immune microenvironment of OS, which includes T lymphocytes, natural killer (NK) cells, TAMs, and myeloid-derived suppressor cells (MDSCs), plays two roles: it helps the body fight cancer and helps it evade immune attack. Conventional immunotherapeutic approaches, including vaccines, cytokine therapy, and immune checkpoint blockade (ICB), have shown limited efficacy in osteosarcoma (OS). Conversely, novel nanoinmunotherapy strategies are being developed to counteract the immunosuppressive TME, trigger immunogenic cell death (ICD), and facilitate targeted delivery to enhance therapeutic outcomes. Genomic alterations, including the loss of *TP53* or *RB1* and the amplification of *MYC* or *MDM2*, interact with metabolic and epigenetic modifications, such as *KDM6B*-driven chromatin remodeling and a metabolic shift toward glycolysis, thereby increasing the propensity for osteosarcoma (OS) metastasis. These intratumoral alterations are influenced by signals emanating from the TME, specifically those generated by tumor-associated macrophages (TAMs), myeloid-derived suppressor cells (MDSCs), and activated platelets. Consequently, these interactions facilitate the dissemination and survival of osteosarcoma cells in distant anatomical sites. Furthermore, pro-migratory signaling pathways, including *PI3K*/*AKT*, *MAPK*, *WNT*/*β-catenin*, and *Rho*/*Rac*, in conjunction with chemokine, integrin networks, such as *CXCL12*/*CXCR4*/*CCR7*, collectively drive these metastatic processes. Rational combination strategies, including ICB with TAM/MDSC modulation, inhibition of chemokine or integrin axes, and nano-immunotherapy, represent promising therapeutic pathways. Future initiatives should emphasize biomarker-driven clinical translation, sophisticated delivery mechanisms, and single-cell/spatial analyses to address IME heterogeneity and surmount therapeutic resistance in OS. *AXL* is a key regulator of OS progression and is strongly associated with a bad prognosis [161]. It is a receptor tyrosine kinase (RTK). In murine models, *AXL* inhibition markedly diminished the pulmonary metastases from MG63.2 cells [162]. Clinical trials of multi-target tyrosine kinase inhibitors (TKIs) have demonstrated that *AXL* inhibition can elicit partial remission in some patients [163]. While these results are encouraging, no clinical trial has yet examined the impact of selective AXL inhibition alone on outcomes in OS patients. Preclinical studies further demonstrate that AXL knockdown inhibits OS cell proliferation and induces apoptosis [164]. AXL overexpression is observed in rapidly spreading, ubiquitously distributed OS cell lines, and blocking it slows tumor cell growth, invasion, and cell spread [161]. Fibroblast growth factor receptor (FGFR) is another RTK frequently overexpressed in OS. FGFRs control many bodily functions, including the development of the nervous system, organ formation, and tissue healing [165]. *FGFR1* is expressed in about 74% of OS samples, making it a very promising target for treatment [166].

### 9.2. Chondrosarcoma (CS)

Chondrosarcoma (CS) is a diverse group of malignant bone tumors characterized by the creation of a cartilaginous matrix [167]. This cancer mainly affects adults and is most common in the axial skeleton, particularly the pelvis and the upper parts of long bones like the femur and humerus [168]. Tumors arise from chondrocytes or their precursors, with most instances classified as conventional central CS [169]. Less common subtypes, such as dedifferentiated and mesenchymal CS, have distinct biological traits and are more aggressive in the clinic, leading to worse outcomes [170]. The risk of metastasis varies significantly based on histologic subtype and grade, as established by classic studies that highlighted the prognostic significance of morphological characteristics [171]. In large-population studies, traditional CS demonstrates a relatively low metastatic rate, with approximately 6% of patients exhibiting metastases at diagnosis [172]. On the other hand, dedifferentiated variants and higher-grade lesions are more likely to spread early [173,174]. When metastasis occurs, the lungs are the most common site, followed by the pleura and skeletal sites (ribs, spine). Nodal spread is not very common. The frequent and early appearance of dedifferentiated and mesenchymal carcinoma highlights the need for precise histological classification. Molecular pathogenesis indicates ongoing genetic and epigenetic changes. Mutations in *IDH1* and *IDH2* are found in about half of the cases of CS151. These mutations result in the oncometabolite D-2-hydroxyglutarate, which inhibits α-ketoglutarate-dependent dioxygenases and alters DNA and histone methylation [175,176]. Several cohorts have reported that *IDH* mutation status is associated with enhanced metastasis-free survival, suggesting that alternative molecular pathways facilitate metastasis in IDH-mutant CS [177]. In addition to IDH, dysregulated Hedgehog signaling and *PI3K*/*AKT* activation are present; however, Smoothened inhibition has not produced significant clinical benefit [178]. Genomic studies have also identified changes in *COL2A1* (type II collagen) and *TP53* in some patients, but we still do not know exactly how these changes help cancer spread [179]. The CS TME is characterized by a dense cartilaginous ECM abundant in collagen and proteoglycans, which impedes drug delivery and transmits pro-invasive signals [180]. *MMP-1*/*-2*/*-13* must change the ECM for invasion to happen, and their levels are linked to how aggressive the cells are [181]. Simultaneous overexpression of lysyl oxidase (*LOX*) enhances collagen cross-linking and matrix stiffening, a change that seems contradictory but can actually help migration by increasing integrin-mediated traction [182,183]. CS is generally hypovascular and hypoxic, which stabilizes *HIF-1α* and starts programs for angiogenesis and migration [184]. Hypoxia also causes the release of extracellular vesicles and exosomes, which can alter the immune response in the area (for example, by promoting macrophage differentiation to an M2 phenotype) and create an immunosuppressive niche through *IL-10* and *TGF-β* [185]. Non-malignant stromal components, including mesenchymal stromal cells and fibroblasts, supply growth factors and matrix-modifying enzymes; elevated microvessel density is associated with tumor grade and metastatic potential [186]. Many signaling pathways work together to control growth, survival, invasion, and resistance to treatment. These include *PI3K*/*AKT*/*mTOR*, *SRC*, and *TGF-β* [187]. Dedifferentiated CS often exhibits further genomic alterations similar to those observed in OS, which intensifies its metastatic characteristics [188]. Bone-tropic interactions, such as *RANKL* signaling, could facilitate seeding within the skeletal system [189]. Because of the rarity of this condition, much of the existing evidence stems from correlational analyses of human tissues and in vitro investigations. However, the data consistently associate elevated MMP expression, *HIF-1α* activity, and unique exosomal RNA signatures with metastatic risk [185]. Proof-of-concept studies indicate that targeting angiogenesis/*HIF-1α* or macrophage polarization may retard progression [190]. Anti-angiogenic tyrosine kinase inhibitors (TKIs), such as pazopanib and regorafenib, have demonstrated limited effectiveness in stabilizing disease progression in advanced CS [191]. Epidemiologically, CS constitutes approximately 20–30% of all malignant bone tumors, ranking as the second most prevalent primary malignant bone neoplasm after OS. CS primarily occurs in adults over 40 years old [192,193], whereas OS is more common in children and adolescents. It is a term that encompasses groups of things with different biology, genetics, and epigenetics. The occurrence of CS, especially atypical cartilaginous tumor (ACT), the low-grade variant, has risen, likely due to an aging population and enhanced diagnostic imaging [194]. Most patients have good outcomes after a wide resection because conventional CS grows slowly and rarely spreads. Nonetheless, advanced, metastatic, or unresectable disease presents a dismal prognosis due to its resistance to chemotherapy and radiotherapy, coupled with a scarcity of effective systemic treatments. Recent studies have underscored a range of genetic and molecular modifications associated with disease advancement and the transition to high-grade or dedifferentiated phenotypes. Some of the most important ones are changes to isocitrate dehydrogenase 1 and 2 (*IDH1*/*2*), an increase in *EPAS1*, which encodes the hypoxia-inducible factor 2-alpha (HIF-2α), and an increase in the *SIRT1*/*HIF-2α* signaling axis. These changes make tumors more aggressive, change their metabolism, and help them adapt to low oxygen levels, which leads to malignant transformation and resistance to treatment. It also delineates the progress and constraints of near-patient preclinical models, as well as the potential of novel therapies targeting cancer stem cell dependencies or employing immunological strategies. Integrative profiling has improved risk stratification. A CS multi-omics signature derived from mRNA, microRNA, and DNA methylation identified high-risk patients in one of the largest genetically characterized cohorts to date, highlighting the combined effects of upregulated cell-cycle programs, silencing of the 14q32 imprinted locus (with downregulation of *miR-154*, *miR-382*, and *miR-384*, previously shown to suppress bone sarcoma growth), and genome-wide hypermethylation induced by *IDH* mutations in driving higher grade and worse prognosis [195]. This stratification framework delineated three favorable-prognosis subgroups-*IDH*^wt^/14q32^high^, *IDH*^mut^/14q32^high^, and *IDH*^wt^/14q32^low^, alongside two intermediate-risk groups (*IDH*^mut^/14q32^low^ and Polif^high^) and a dedifferentiated cohort (14q32^low^/Prolif^high^) correlated with the most adverse clinical outcomes [188]. These results highlight the importance of comprehensive molecular profiling beyond *IDH* mutation status alone and help resolve previously noted discrepancies in prognostic classification. Single-cell RNA sequencing identified four signatures based on proliferation, stromal, or leukocyte-related genes. High-grade and dedifferentiated tumors exhibited elevated proliferation indices, with an immunosuppression index distinguishing the dedifferentiated group, while a “active immune response” index characterized low-growing tumors [179]. The ER-stress regulators *DDIT3*/*CHOP* and *HSPA5* were identified as survival indicators in standard central CS, with increased expression associated with adverse outcomes. In CS PDX models, inducing ER stress accelerated growth, while its inhibition hindered progression, establishing ER stress as a therapeutic target. Epigenetic therapies are mechanistically justified by *IDH1*/*2*-induced elevations in DNA and histone methylation; however, *IDH*-mutated dedifferentiated chronic lymphocytic leukemia (CS) displays reduced hypermethylation and distinct loci compared with IDH-mutated conventional CS [193]. The FDA has approved DNA hypomethylating agents (like 5-aza-2′-deoxycytidine and decitabine) and HDAC inhibitors (like vorinostat, romidepsin, belinostat, and Panobinostat) for use in hematologic malignancies. In preclinical studies, 5-aza plus vorinostat was more effective than either agent alone in vitro and in JJ012 xenografts. It also triggered more DNA-damage responses and activated interferon-stimulated genes (including *PD-L1*). It activated the innate immune system [66,196]. A phase II trial of guadecitabine (DNMT inhibitor) and belinostat (HDAC inhibitor) in unresectable or metastatic conventional CS (NCT04340843) failed to achieve the primary overall response rate (ORR) endpoint [197]. The next steps involve assessing the efficacy of epigenetic therapy in conjunction with immune checkpoint inhibitors and chemotherapy. In general, CS metastasis occurs when intrinsic factors (such as *IDH* mutations, *COL2A1*/*TP53* changes, and pathway dysregulation) and extrinsic factors (such as ECM remodeling, hypoxia, and immune reprogramming) converge. The fact that *IDH*-mutant tumors may have better metastasis-free survival underscores molecular heterogeneity and suggests distinct metastatic circuits within subgroups [179]. Current research priorities focus on elucidating the metastatic mechanisms underlying *IDH*-wild-type CS and on developing physiologically relevant preclinical models [198].

### 9.3. Liposarcoma (LPS)

Liposarcoma (LPS), a diverse group of malignant tumors made of fat cells, includes many histological subtypes. These subtypes differ greatly in their genetic causes, clinical behavior, and response to treatment [199]. The primary variants include well-differentiated LPS (WDLS), dedifferentiated LPS (DDLS), myxoid/round-cell LPS (MLPS), and pleomorphic PLPS, each characterized by unique morphology, genetic alterations, and clinical features [200]. In older adults, WDLS and DDLS are most likely to occur in the deep soft tissues of the extremities or retroperitoneum. These tumors represent a biological progression, where dedifferentiation leads to the development of DDLS from pre-existing WDLS. MLPS typically involves the limbs of younger adults, and its defining features include abundant myxoid stroma and a branching vascular pattern. Its molecular signature is the *FUS-DDIT3* (TLS-CHOP) fusion oncogene produced by the t(12;16)(q13;p11) chromosomal translocation [201]. PLPS is the rarest but most aggressive subtype, usually found in the limbs, characterized by a high-grade pleomorphic appearance and various types of fat cells [202]. The risk of metastasis varies significantly across subtypes [203]. WDLS seldom metastasizes, and mortality is generally associated with local recurrence, especially in retroperitoneal tumors where complete resection is difficult. DDLS, on the other hand, is highly aggressive and can spread to other parts of the body in up to 30% of cases, most often to the lungs [204]. Conversely, MLPS tends to metastasize beyond the pulmonary system, frequently affecting the osseous structures, vertebral column, and retroperitoneal soft tissues; approximately 17% of cases present with skeletal metastases [205]. PLPS, in contrast, demonstrates features similar to those of undifferentiated pleomorphic sarcoma, such as rapid dissemination, pulmonary metastases, and poor survival [206]. Molecular pathogenesis is specific to subtypes. WDLS and DDLS are marked by a steady increase in chromosome 12q13–15, which includes *MDM2* (almost always) and *CDK4* (often). These two genes work together to cause cancer by turning off p53-mediated checkpoints and speeding up the cell cycle [207]. The *FUS-DDIT3* fusion is what makes MLPS. It stops adipocytic differentiation and causes tumors to grow. Some isoforms are linked to a higher risk of skeletal metastasis [208]. More mutations, like *TP53* and *RB1*, may build up over time and are linked to resistance to treatment and the ability to spread to other parts of the body [209]. The TME also changes how metastasis works. DDLS frequently arises within a dense, fibrotic stroma populated by CAFs, which remodel the ECM and facilitate tumor growth [210]. Exosomes from tumors that carry oncogenic RNAs and microRNAs help form pre-metastatic niches, especially in the lungs [211]. Immune profiling of DDLS reveals inflamed and non-inflamed subtypes. The different types of T-cell infiltration and macrophage polarization affect prognosis and treatment response [212]. The *CXCR4-CXCL12* chemokine axis is very important for guiding myxoid LPS cells to secondary organs that are rich in *CXCL12*. There, ligand–receptor gradients increase cells’ ability to move (chemotaxis) and survive during dissemination. When tumor-derived angiogenic mediators like *VEGFA*, *ANGPT2*, and HIF-regulated cytokines are disseminated, they promote the formation of new blood vessels and alter the local stroma, making it easier for metastases to grow [213]. More generally, receptor tyrosine kinase pathways, such as PDGFR and IGF, along with the activation of PI3K/AKT/mTOR signaling, support proliferation, invasion, and survival across various LPS subtypes [214]. In clinical practice, these biological differences lead to different approaches to monitoring and treating patients. For instance, MLPS patients are now advised to have regular spinal imaging because they are at a high risk of bone metastases [215]. In the context of desmoplastic small round cell tumors (DDLS), the efficacy of immune checkpoint blockade has proven limited, thereby underscoring the critical need for predictive biomarkers and combination therapies targeting *TGF-β*, angiogenesis, or immune evasion pathways [216]. A confluence of genetic determinants unique to each subtype, the surrounding cellular microenvironment, and intricate signaling cascades regulates the dissemination of LPS. The disparate patterns of MLPS and DDLS dissemination necessitate a cautious approach to both monitoring and therapeutic interventions. Future research endeavors should prioritize translating genomic findings into targeted therapies, refining biomarker-driven patient stratification, and developing preclinical models that faithfully replicate metastatic niches to advance novel treatments [217]. About 1% of all cancerous tumors are sarcomas. LPS, a type of soft tissue sarcoma, is the most common histological type, accounting for 15–20% of cases. LPS arises from adipocytic differentiation and primarily presents in the lower extremities or retroperitoneum [218]. It is classified according to immunohistochemical profiles, cellular morphology, and related genetic alterations220. Each subtype displays unique biological characteristics, molecular signatures, and pharmacological sensitivities [219].

Surgical intervention remains the cornerstone of therapeutic approaches for liposarcoma (LPS). The anatomical positioning of the tumor significantly influences both the feasibility of surgical resection and the patient’s prognosis. Retroperitoneal LPS often manifests asymptomatically within the expansive retroperitoneal space, thereby challenging complete resection and contributing to a high recurrence rate. Given the physical and psychological toll of repeated surgical procedures, the success of the initial surgical intervention is critical for determining the long-term outcome. Systemic therapy for LPS predominantly utilizes anthracycline-based chemotherapy, sometimes in conjunction with other cytotoxic agents to enhance efficacy, albeit at the cost of heightened toxicity [220]. Nonetheless, advancements in molecularly targeted and immunotherapeutic strategies are broadening the treatment spectrum; most LPS subtypes do not respond well to standard chemotherapies. This review outlines the clinicopathologic characteristics, molecular pathogenesis, and contemporary management strategies of LPS subtypes, with a focus on novel approaches, including targeted therapies and immunotherapies. As our understanding of genetics and molecular biology advances, we can expect better-designed treatments to yield better clinical outcomes. The increased sensitivity of MLPS to drugs, compared to other subtypes, is a promising finding for drug development. New drugs, such as anthracycline derivatives, TKIs, marine-derived compounds, and immune modulators, are showing different levels of benefit, which supports the use of combination strategies in the future.

### 9.4. Melanoma

**Figure 3:**
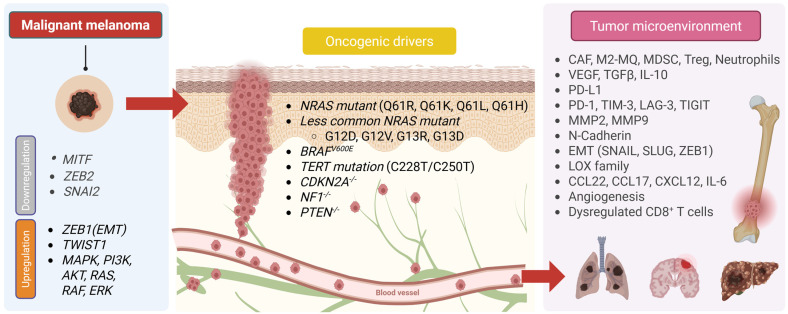
Integrated oncogenic and microenvironmental mechanisms driving malignant melanoma metastasis. Malignant melanoma progression and metastatic dissemination are orchestrated by the convergence of tumor-intrinsic oncogenic alterations and TME–derived signals that collectively promote phenotypic plasticity, invasion, and immune evasion. Common oncogenic drivers, including activating *NRAS* mutations (Q61 variants) and *BRAF^V600E^*, together with loss of tumor suppressors such as *PTEN*, *CDKN2A*, and *NF1*, constitutively activate MAPK-ERK and PI3K-AKT signaling pathways, enhancing melanoma cell survival, proliferation, and motility. These pathways induce a transcriptional reprogramming characterized by upregulation of EMT-associated transcription factors (*ZEB1*, *SNAI2*/SLUG, *TWIST1*) and concomitant downregulation of melanocytic differentiation programs (e.g., *MITF*) and epithelial adhesion molecules, facilitating an EMT-like, invasive phenotype. Tumor–stroma interactions further reinforce this transition, as CAFs and infiltrating myeloid populations (M2-like macrophages, MDSCs, Tregs, neutrophils) secrete cytokines and growth factors (VEGF, TGF-β, IL-6, IL-10) that promote matrix remodeling (*MMP2*, *MMP9*), angiogenesis, immune checkpoint engagement (PD-1/PD-L1, TIM-3, LAG-3, TIGIT), and suppression of CD8^+^ T-cell function. Chemokine gradients (CCL22, CCL17, CXCL12) and adhesion changes (N-cadherin induction) facilitate tumor cell intravasation, survival in circulation, and colonization of distant organs, including lung, brain, bone, and liver. Take-home message: melanoma metastasis is not driven by a single pathway but emerges from the coordinated activation of oncogenic signaling, EMT-driven plasticity, and an immunosuppressive, pro-metastatic microenvironment, highlighting the need for therapeutic strategies that simultaneously target tumor cell–intrinsic programs and microenvironmental dependencies.

Melanoma is a highly aggressive malignancy originating from neural crest-derived melanocytes found in cutaneous, mucosal, and ocular regions; cutaneous melanoma is the predominant subtype [221]. Histologically, lesions range from radial growth-phase tumors limited to the epidermis to vertically invasive tumors that penetrate the basement membrane; cells may be pigmented or amelanotic, but the hallmark is invasive growth beyond the site of origin [222]. Melanoma is clinically significant because it spreads to other parts of the body early and often through both lymphatic and hematogenous pathways, which explains why it has such a big effect on skin cancer deaths [223]. The initial phase of dissemination typically entails migration via dermal lymphatics to regional lymph nodes; consequently, nodal status serves as a crucial prognostic indicator, informing staging and management (Figure 3) [224]. After that, the disease spreads through the blood to the lungs, liver, brain, bones, and skin/subcutaneous tissue. Brain metastases happen in a lot of advanced cases and have a big effect on survival and quality of life [225]. In-transit metastasis, in which tumor deposits form between the primary site and the regional basin, is a unique sign of lymphatic dissemination. This is because melanoma has lymphotropism and is hard to treat because it spreads to multiple places [226]. At the molecular level, metastatic competence is based on well-defined oncogenic changes that occur repeatedly [227]. *BRAF V600* mutations (most often V600E) turn on MAPK/ERK signaling all the time and are found in about half of cutaneous melanomas. *NRAS* mutations (about 15–20%) activate both the MAPK and PI3K/AKT pathways [228]. Disruption of tumor suppressors like *NF1*, *PTEN*, and *CDKN2A* destabilizes growth and survival signaling [229]. Furthermore, *TERT* promoter mutations, which are common and enhance telomerase activity and cellular immortality, contribute to accelerated progression [230]. Melanoma cells utilize the MAPK, PI3K/AKT, WNT/β-catenin, and *NF-κB* signaling pathways to regulate growth, survival, immune evasion, and metastasis [231]. Invasion and dissemination are contingent upon dynamic alterations in adhesion and motility. Melanoma cells often downregulate E-cadherin and upregulate N-cadherin, thereby promoting detachment from keratinocyte constraints and augmenting interactions with stromal and endothelial components [232]. *MMP-2* and *MMP-9* facilitate the degradation of the basement membrane and suppression, and invasion [233]. The immune environment significantly influences prognosis; specifically, a high density of CD8^+^ T cells in the appropriate locations correlates with favorable outcomes, whereas an abundance of regulatory T cells and M2 macrophages is indicative of poor prognosis [234]. Melanoma promotes lymphangiogenesis within lymph nodes, thereby expanding lymphatic channels and promoting dissemination [235]. Immune evasion is a critical factor in the disease’s successful spread. The upregulation of *PD-L1* on melanoma cells engages *PD-1* on T cells, thereby inhibiting effector function and promoting tumor progression, a mechanism that is now effectively targeted in clinical practice [236]. The “immunotherapy revolution” highlights the significance of the TME. Checkpoint inhibitors have demonstrably improved the prognosis for numerous patients, although primary and acquired resistance remain prevalent and necessitate biomarker-driven combination therapies [237]. Preclinical models that closely mimic human disease have provided insights into the biology of metastasis and therapeutic resistance. Genetically engineered mouse models, like *BRAF^V600E^* with *PTEN*^−/−^, mimic spontaneous metastasis. B16 syngeneic and human xenograft models build on these results, especially for lung/brain spread and treatment evaluation [238]. Immune infiltration patterns and driver genotypes correlate with prognosis and therapeutic response, elucidating prevalent resistance mechanisms via secondary mutations or pathway reconfiguration [239]. Overall, melanoma metastasis results from the interaction of well-known oncogenic drivers (such as mutations in BRAF, NRAS, and the TERT promoter), changes in adhesion and motility that help the cancer spread, chemokine-directed organotropism, and an immune-modulatory TME [240]. More investigation is needed to verify the factors influencing immune-infiltration heterogeneity, developing predictive biomarkers for targeted and immunotherapies, and devising logical combinations that concurrently tackle tumor-intrinsic pathways and TME-mediated resistance [241]. Single-cell/spatial multi-omics, liquid biopsy monitoring, and next-generation, immune-competent preclinical models that better mimic human metastatic disease [242] will speed up progress.

### 9.5. Hepatocellular Carcinoma (HCC)

Hepatocellular carcinoma (HCC), the most common type of primary liver cancer, develops from liver cells, usually in people with long-term liver disease and cirrhosis [243]. Most cases arise from chronic hepatic inflammation caused by hepatitis B or C infection, alcohol-related liver disease, or non-alcoholic steatohepatitis [244]. Histologically, HCC exhibits trabecular and pseudoglandular proliferation of atypical hepatocytes accompanied by architectural distortion. Fibrolamellar HCC is a unique type that happens in younger patients who do not have cirrhosis. It has its own molecular characteristics and clinical behavior [245]. Clinically, HCC is widespread worldwide and is marked by aggressive biology and early metastatic potential, leading to unfavorable survival outcomes [246]. HCC metastasis indicates its intrahepatic origin and significant vascular invasiveness into the portal and hepatic veins, which promote hematogenous dissemination and are associated with a poor prognosis [247]. The lung is the most common place outside the liver, followed by the lymph nodes in the abdomen, and the spread of cancer through the peritoneum. Bone metastases, which usually involve the axial skeleton, often cause severe pain and fractures [248]. Other targets include the diaphragm, brain, adrenal glands, and pleural surfaces [248]. Extensive clinical studies consistently indicate a decreasing prevalence of involvement in the lung, peritoneum, bone, spleen, adrenal glands, brain, pleura, and kidneys [249]. Brain metastases are infrequent but generally occur late and indicate a poor prognosis for survival [250]. At the molecular level, chronic inflammatory injury creates a mutagenic environment that promotes cancer growth and the spread of cancer cells to other parts of the body [251]. Recurrent alterations include *TERT* promoter mutations that reactivate telomerase [252], *TP53* mutations that hinder genomic surveillance, and *CTNNB1* (β-catenin) mutations that promote Wnt pathway activation [253,254]. Other factors that sustain angiogenesis and promote metastatic colonization include *MET* activation and widespread upregulation of the VEGF pathway. Metastatic dissemination entails epithelial–mesenchymal transition, facilitated by *Snail*/*Zeb* factors and often triggered by TGF-β- rich inflammation, while matrix metalloproteinases, including MMP-9, degrade the ECM to facilitate invasion [255]. HCC develops in a microenvironment characterized by cirrhosis, fibrosis, and inflammation, which is intrinsically immunosuppressive [256]. Activated hepatic stellate cells and portal fibroblasts generate a collagen-rich matrix and induce tissue stiffening, which influences tumor behavior and hinders drug penetration [257]. CAFs alter ECM, release VEGF, PDGF, and chemokines, and keep the immune system out by signaling through IL-6 and TGF-β and building physical barriers [258]. Macrophages, particularly M2-like subsets, play roles in immune suppression, angiogenesis, and matrix remodeling [259]. VEGF-induced endothelial hyperproliferation results in disordered vasculature that promotes intravasation and is associated with vascular invasion [260]. These interactions between stromal and immune cells, along with oncogenic pathways like PI3K/AKT activation, *PTEN* loss, FGF/IGF signaling, and *PD-L1* upregulation, work together to support tumor growth, invasion, and immune evasion [261,262,263]. Molecular profiling has helped identify metastasis-related signatures, including exosome-derived miRNAs that affect distant microenvironments and promote the formation of metastatic niches [264]. In clinical practice, multiple-organ metastases frequently occur after surgical resection, with the lung, bone, and peritoneum being the most commonly affected sites [265]. In general, HCC metastasis is driven by chronic inflammation-induced mutagenesis, including mutations in TERT, TP53, and CTNNB1.

### 9.6. Glioblastoma (GBM)

**Figure 4:**
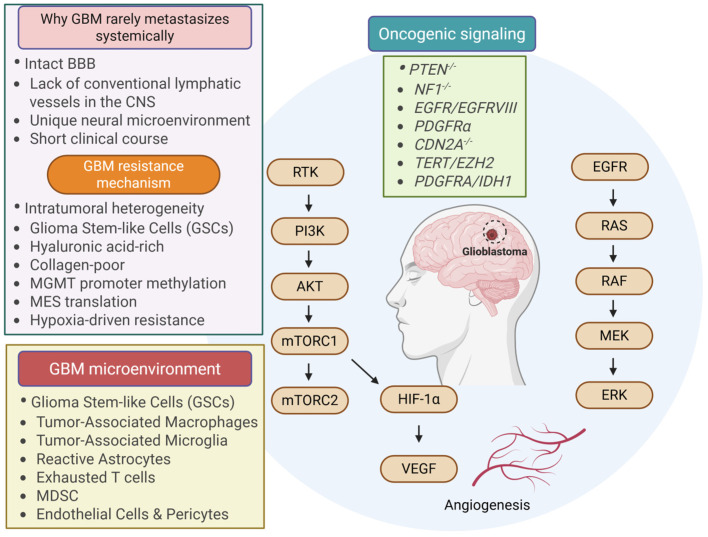
Integrated oncogenic signaling, microenvironmental interactions, and resistance mechanisms in GBM. This schematic summarizes key biological features that underlie GBM progression and therapeutic failure. GBM rarely metastasizes outside the central nervous system due to the restrictive blood–brain barrier, absence of conventional lymphatic drainage, and a unique neural microenvironment that constrains systemic dissemination. Despite this confinement, GBM exhibits profound therapeutic resistance driven by intratumoral heterogeneity, the presence of therapy-resistant glioma stem-like cells, limited drug penetration, and adaptive responses to hypoxia and metabolic stress. The TME is enriched with immunosuppressive myeloid populations, reactive glial cells, dysfunctional vasculature, and exhausted T cells, collectively promoting immune evasion and local recurrence. Concurrently, recurrent genetic alterations and oncogenic signaling pathways—most prominently RTK-driven PI3K–AKT–mTOR and RAS–RAF–MEK–ERK cascades, enhance tumor cell survival, proliferation, and angiogenesis through HIF-1α–VEGF signaling. These interconnected tumor-intrinsic and microenvironmental mechanisms explain why GBM remains locally aggressive and highly treatment-refractory despite its limited capacity for systemic metastasis.

GBM is the most aggressive primary cancer of the central nervous system. The 2021 update of the World Health Organization classification [266] states that it is a WHO grade 4 astrocytic tumor. GBM, which mainly arises from glial cells, primarily astrocytes, is characterized by pseudopalisading necrosis and microvascular proliferation under a microscope. These traits indicate a malignant biology and set it apart from lower-grade gliomas [267]. In clinical settings, GBM is distinguished by its invasive nature, rapid recurrence, and consistently unfavorable prognosis, notwithstanding aggressive multimodal treatments. These treatments typically involve maximal surgical resection, radiotherapy, and temozolomide chemotherapy; nevertheless, median survival times rarely exceed 15 months [268]. Unlike numerous other systemic malignancies, GBM infrequently metastasizes beyond the central nervous system (Figure 4). This unique confinement is due to the protective blood–brain barrier, the absence of a traditional lymphatic system, the specialized neural microenvironment, and the short clinical course that limits the time for systemic dissemination [269]. Unlike most solid tumors, GBM does not metastasize through classical hematogenous routes. Instead, its lethality arises from extensive local invasion within the brain parenchyma. Tumor cells infiltrate along white matter tracts, perivascular spaces, and cerebrospinal fluid pathways, occasionally giving rise to leptomeningeal or spinal “drop” metastases [270]. True extracranial dissemination is exceedingly rare and is most often linked to iatrogenic disruption, such as craniotomy or ventricular shunting, which can permit tumor cell escape [269]. Thus, GBM progression is defined less by distant organ colonization than by diffuse, infiltrative growth that renders complete surgical eradication impossible. At the molecular level, GBM exhibits striking heterogeneity. Verhaak and his team used transcriptomic analyses to identify three main expression patterns: classical, mesenchymal, and proneural [271]. Classical tumors often harbor *EGFR* amplification or mutations, including the *EGFRvIII* variant, which drives continued activation of the Ras/MAPK and PI3K/AKT signaling pathways [271,272]. A highly invasive phenotype driven by *NF1* loss and increased TGF-β signaling is characteristic of mesenchymal GBMs [273]. In contrast, proneural tumors frequently harbor *PDGFRA* amplification or IDH1 mutations; the latter are characteristic of IDH-mutant gliomas and are associated with the CpG island methylator phenotype (G-CIMP), frequent *MGMT* promoter methylation, and comparatively favorable clinical outcomes [268,274]. Importantly, subsequent single-cell RNA sequencing studies demonstrated that individual GBM tumors rarely conform to a single transcriptional category. Instead, cells corresponding to multiple expression states coexist within the same lesion, indicating that these subtypes reflect dynamic transcriptional programs shaped by microenvironmental cues and therapeutic pressure rather than fixed lineages [275,276]. The heterogeneity and high plasticity of GBM cells highlight the biological complexity of this disease, as initially captured by the Verhaak transcriptional classification system. Invasion is further facilitated by integrin-mediated adhesion and by ECM remodeling. Interactions between the α6β1 integrin and laminin enable tumor cell migration along vascular pathways. Matrix metalloproteinases, such as MMP-2 and MMP-9, degrade type IV collagen, which reshapes the neural ECM [277]. Consistent with this invasive behavior, glioma cells show considerable motility plasticity, switching between elongated, protease-dependent mesenchymal migration and rounded, deformable amoeboid movement in response to environmental constraints [278]. This flexibility allows it to spread through both dense and flexible parts of the brain. Hypoxia further increases invasiveness by stabilizing HIF-1α, which in turn increases VEGF and EMT transcription factors such as ZEB1. This leads to angiogenesis and mesenchymal transition [279]. Tumor-associated macrophages and microglia reportedly comprise up to fifty percent of the tumor mass and frequently exhibit an M2-like phenotype, secreting IL-10 and TGF-β to inhibit T-cell activity [233]. Single-cell analyses have elucidated a range of TAM states and their reciprocal interactions with glioma cells that facilitate invasion and angiogenesis [280]. In GBM, the presence of tumor-infiltrating lymphocytes is often limited, and these cells frequently demonstrate functional exhaustion, which diminishes the effectiveness of immune checkpoint blockade [281]. Simultaneously, reactive astrocytes and endothelial cells actively contribute to a tumor-supportive microenvironment by releasing growth factors and cytokines that foster immune suppression, invasion, and tumor survival [282]. These characteristics, in concert, create a microenvironment that protects GBM from immune-mediated destruction while facilitating its continuous growth. Aberrant activation of *EGFR* amplification, *PTEN* loss, and *TP53* mutation are among the most prevalent genomic alterations in this malignancy [283]. Constitutive epidermal growth factor receptor (EGFR) signaling activates both the mitogen-activated protein kinase (MAPK) and phosphoinositide 3-kinase (PI3K)/AKT pathways, thus facilitating cellular proliferation, survival, and metabolic adaptation [284]. Furthermore, mTORC2-mediated phosphorylation of AKT specifically converges on the MNK-dependent phosphorylation of eIF4E, exacerbating invasive behavior and conferring resistance to therapeutic interventions [285]. The pharmacologic MNK inhibitor tomivosertib reduces eIF4E activation, suppresses angiogenic signaling, and enhances the sensitivity of GBM cells [286]. Epigenetic dysregulation plays a major role in the diversity and adaptability of GBM. Phenotypic plasticity, recognized as a nascent characteristic of GBM, is closely associated with epigenetic reprogramming [287]. Patterns of DNA methylation influence both tumor biology and therapeutic responsiveness. For example, MGMT promoter methylation predicts improved responses to temozolomide, while the G-CIMP phenotype, most commonly observed in IDH-mutant gliomas, defines a distinct epigenetic and prognostic subgroup [288]. At the chromatin level, EZH2-mediated H3K27 methylation inhibits tumor suppressors like PTEN and collaborates with PI3K signaling to promote oncogenic transcription [289]. By deacetylating α-tubulin and stabilizing EGFR [290], HDAC6 helps glioma cells grow and move. Preclinical investigations have demonstrated that pharmacological agents targeting HDAC6 or EZH2 can diminish cellular invasive capacity. Conversely, clinical applications of broad-spectrum HDAC inhibitors or agents that reverse DNA methylation have yielded limited therapeutic success. This underscores the need for more precisely targeted epigenetic therapeutics capable of crossing the blood–brain barrier. The emergence of treatment resistance in GBM multiforme is attributable to a confluence of genetic heterogeneity, the capacity of cells to adopt stem cell-like characteristics, and the influence of protective elements within the TME [291]. Recurrence usually occurs because of pre-existing resistant subclones that survived chemoradiation. Recurrent tumors often exhibit mesenchymal enrichment and therapy-induced changes [292]. Glioma stem-like cells remain dormant, facilitate DNA repair by activating ATM/ATR, and replenish tumors post-therapy [293]. These cells also increase the activity of efflux transporters and anti-apoptotic genes, which makes multidrug resistance even stronger. These mechanisms elucidate the reasons for the recurrent failures of single-pathway targeted therapies. EGFR inhibitors and anti-angiogenic agents like bevacizumab produce temporary responses but lack a sustained survival advantage [294]. Recent progress is changing the way we treat diseases. Researchers are exploring new methods to deliver drugs into the body, including convection-enhanced drug infusion, nanoparticles, and peptide-mediated blood–brain barrier penetration [295]. Non-pharmacologic modalities, especially tumor-treating fields, have improved median survival when combined with temozolomide, underscoring the importance of integrating novel technologies [296]. PD-1 blockade, multi-antigen vaccines, and regionally administered CAR T cells targeting IL13Rα2 or HER2 can trigger an anti-tumor immune response even in the immunosuppressive environment of GBM [297]. Oncolytic virotherapy has demonstrated potential; the HSV-1-derived Delytact (G47Δ) attained sustained disease control in a cohort of patients with recurrent GBM [298], representing the inaugural approved virotherapy for malignant glioma. Even with these small improvements, GBM is still a model of an adaptive cancer. Its confinement in the brain conceals a remarkable capacity to exploit the microenvironment, evade treatment, and recur. For future success, we will need to use combinations of treatments that target oncogenic signaling, epigenetic plasticity, and immune suppression simultaneously, while also making it easier for drugs to cross the blood-brain barrier. Combining genomic, epigenomic, and spatial single-cell analyses is helping us better understand how different cell types in a tumor interact and how to develop combination therapies [299]. With ongoing progress in multi-omics profiling, immunoengineering, and precision drug delivery, the enduring therapeutic stalemate of GBM may finally commence to dissolve.

### 9.7. Breast Tumor

Breast cancer metastasis is a slow and complex process, only completed by a small number of tumor cells [300]. These metastasis-initiating cells (MICs) have stem-like properties and a high degree of phenotypic plasticity. This allows them to survive systemic stress, avoid immune responses, and adapt to different tissue environments [301]. Epithelial–mesenchymal transition (EMT) is a key factor in the plasticity of MICs. In breast tumors, transcription factors such as *SNAIL*, *ZEB1*, and *PRRX1* regulate this process.

**Figure 5:**
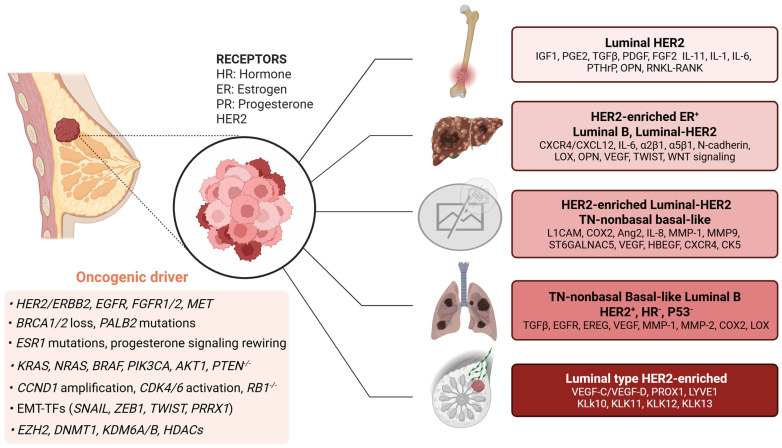
Receptor status–defined oncogenic programs and organ-specific metastatic tropism in breast cancer. Breast cancer subtypes are defined by hormone receptor (HR; ER/PR) and *HER2* expression, which together shape lineage identity, oncogenic dependencies, and metastatic behavior. Left, major oncogenic drivers shared across subtypes include receptor tyrosine kinase activation (*HER2*/*ERBB2*, *EGFR*, *FGFR1*/*2*, *MET*), DNA repair defects (*BRCA1*/*2*, *PALB2*), *ESR1* mutations and hormone signaling rewiring, and activating alterations in *KRAS*/*NRAS*/*BRAF–PI3K–AKT* signaling with concurrent *PTEN* loss. Additional layers of tumor progression involve cell-cycle dysregulation (*CCND1* amplification, *CDK4/6* activation, *RB1* loss), EMT transcriptional programs (*SNAIL*, *ZEB1*, *TWIST*, *PRRX1*), and epigenetic regulators (*EZH2*, *DNMT1*, *KDM6A*/*B*, *HDACs*), collectively enabling tumor survival, plasticity, invasion, and dissemination. Right, breast cancer subtypes exhibit distinct ligand, cytokine, and adhesion signatures that dictate organ-specific metastasis. Luminal HER2 tumors preferentially activate bone-tropic pathways through IGF-1, PGE2, TGF-β, PDGF, FGF2, IL-1/6/11, PTHrP, OPN, and RANKL–RANK signaling. HER2-enriched ER^+^/Luminal B/Luminal-HER2 tumors express CXCR4–CXCL12, integrins (α2β1, α5β1), N-cadherin, LOX, VEGF, TWIST, and WNT signaling components, promoting liver and bone colonization. HER2-enriched Luminal-HER2 and TN-nonbasal basal-like tumors display L1CAM, COX2, ANG2, IL-8, MMP-1/10/12, ST6GALNAC5, VEGF, HBEGF, CXCR4, and CKS, facilitating rapid lung and brain metastasis. TN-nonbasal basal-like Luminal B and HER2^+^/HR^−^/p53-mutant tumors exhibit highly inflammatory and proteolytic profiles (TGF-β, EREG, VEGF, MMP-12, COX2, LOX) associated with aggressive visceral and pulmonary spread. Luminal-type HER2-enriched tumors additionally upregulate VEGF-C/VEGF-D, PROX1, LYVE1, and kallikreins (KLK10–13), indicating lymphangiogenic and HER2-driven metastatic programs. Breast cancer metastasis is not stochastic but is governed by receptor-defined oncogenic circuits that program subtype-specific cytokine, adhesion, and epigenetic states, thereby directing predictable organ-selective metastatic routes.

These epithelial–mesenchymal transition (EMT) transcription factors (EMT-TFs) downregulate epithelial markers, such as *E-cadherin*, while simultaneously upregulating mesenchymal genes, thereby conferring both motility and stemness to the affected cells (Figure 5) [301]. The removal of the EMT inducer *Prrx1* has been demonstrated to promote MET and metastatic colonization within breast cancer models, thereby highlighting the dynamic and reversible characteristics inherent to the EMT program [302]. Furthermore, the TME significantly influences EMT activation in breast cancer. For instance, CAFs release TGF-β1 and other factors that trigger EMT [303]. The TME has a big effect on EMT activation in breast cancer. For example, CAFs secrete TGF-β1 and other substances that induce EMT. On the other hand, TAMs release cytokines such as CCL18 and IL-6, which help cells invade and spread [304]. For example, CCL18 from TAM starts a signaling cascade through PITPNM3/ANXA2 that turns on PI3K/Akt/GSK3β signaling and raises the level of *Snail* [305]. In the same way, IL-6 secreted by TAMs activates the JAK2/STAT3 pathway in breast cancer cells, increasing EMT-TF expression and conferring stem-like, therapy-resistant characteristics [43]. These microenvironmental signals work with cancer-intrinsic EMT programs to produce cells that are highly mobile and resistant to treatment. To leave the primary site, breast cancer cells need to break through the surrounding stroma and basement membranes. They do this primarily by increasing MMP levels, especially MMP-2, MMP-9, and the membrane-anchored MT1-MMP (MMP-14), which degrade type IV collagen and other ECM components [306]. High levels of MMP-2/9 activity are strongly linked to invasive behavior and the spread of cancer to other parts of the body. MT1-MMP, on the other hand, is a key effector that activates pro-MMP-2, boosting proteolytic cascades at the invasive front [307]. Once in circulation, breast cancer cells endure shear stress and immune surveillance by moving as circulating tumor cell (CTC) clusters. These clusters are about 23–50 times more likely to spread than single CTCs [308]. Platelet “cloaking” of circulating tumor cells (CTCs) increases the ability of the immune system to avoid detection while also promoting microvascular arrest and vascular loading [309]. Hypoxic conditions enhance this process by increasing CTC-platelet aggregation, thereby creating a temporary protective niche during hematologenous transit [310]. After extravasation, DTCs that spread to distant organs, such as the lungs and additional bone marrow, can enter a dormancy bottleneck, where they can remain dormant for years. Microenvironmental remodeling often triggers reactivation from dormancy. For example, the neutrophil extracellular trap (NET)-mediated cleavage of laminin produces bioactive fragments that activate b1-integrin-YAP signaling, ultimately promoting metastatic outgrowth [311]. Organ-specific “seed and soil” interactions control metastatic growth. Breast cancer exhibits specific levels of *COX2* (PTGS2) and *MMP1*, which facilitate vascular remodeling and extravasation [312]. In brain metastasis, breast cancer cells aberrantly express *ST6GALNAC5*, facilitating their penetration of the blood–brain barrier [313]. Blocking COX2 or MMP1 stops lung metastasis, and knocking down *ST6GALNAC5* stops brain colonization. Invasive lobular carcinoma (ILC), characterized by the loss of E-cadherin, exhibits distinct metastatic pathways and increased susceptibility to IGF1R pathway inhibition [314]. Metastatic colonies change how they use energy in different organs. Breast cancer cells that preferentially metastasize to bone depend on glycolysis and lactate-driven osteolytic remodeling [315]. Brain metastases adapt to a glutamine-rich environment by enhancing glutamine anaplerosis and metabolic plasticity [316]. EMT-high cells exhibit a distinct susceptibility: ZEB1-mediated ferroptosis sensitivity via the repression of SCD1 and the accumulation of polyunsaturated lipids [317]. New ways to treat people use these ideas. Blocking both TGF-β and CD73 reverses EMT and improves the efficacy of immuno- therapy [318]. Inducing ferroptosis is another way to target cells that initiate metastasis by driving EMT [319]. ENPP1, an enzyme that breaks down cGAMP, promotes metastasis to the brain and bones and stops STING signaling. It is a promising target for treatment [320]. Hedgehog-GLI signaling facilitates bone colonization and resistance to endocrine therapy, while SMO/GLI inhibitors have demonstrated preclinical efficacy [321]. A more in-depth understanding of how these processes work is now being combined with new treatments targeting EMT circuits, metabolic weaknesses such as ferroptosis, pro-metastatic enzymes such as ENPP1, and developmental pathways such as Hedgehog [322]. This gives hope for breast cancer-specific treatments that can get rid of dormant seeds and stop the deadly spread of metastases [322]. The ongoing challenge is to turn these discoveries into safe and effective treatments that stop the spread of cancer and improve survival in advanced breast cancer.

## 10. Emerging Therapeutic Strategies for Metastatic Disease

Current therapeutic advances are enhancing our understanding of tumor metastasis and the regulation of the TME. Therapeutic interventions targeting epidermal growth factor receptor (EGFR) and anaplastic lymphoma kinase (ALK) have significantly improved the treatment of metastatic lung cancer by interfering with signaling pathways essential to cancer progression [323]. This targeted approach acknowledges that metastasis is driven by specific biological processes, such as epithelial–mesenchymal transition (EMT), the establishment of pre-metastatic niches, and immune evasion, all of which are amenable to selective targeting [324]. Immune checkpoint inhibitors (ICIs) remain foundational therapies in melanoma and lung cancer by restoring T-cell effector function [325]. However, many metastatic lesions remain immunologically “cold,” lacking effective dendritic-cell priming and T-cell infiltration. To overcome the resistance mechanism, combination immunotherapies are being actively investigated. Therapeutic cancer vaccines enhance neoantigen recognition when combined with PD-1 blockade [326]. At the same time, CD40 and OX40 agonists promote dendritic-cell activation and T-cell costimulation [327], Adoptive cell therapies further extend immune-mediated control, with tumor-infiltrating lymphocyte (TIL) therapy producing durable responses in checkpoint-refractory melanoma and next-generation “armored” CAR-T cells engineered to secrete IL-12 or IL-15 to counteract suppressive solid TMEs [328], Also, reprogramming immunosuppressive M2 macrophages toward inflammatory M1 states and reducing metastatic burden in preclinical models [329]. However, for metastasis suppression and targeting tumor cells to be effective, it is necessary to abolish the “soil” that nurtures metastatic cells. This approach for ablating metastasis involves targeting the “soil” components: either PEGPH20, which removes hyaluronic acid and creates space for increased penetration of anticancer therapies, or fibroblast activation protein, which abolishes fibroblast-rich metastatic sites [330]. In bone metastasis, targeting *RANKL* by denusomab prevents bone-related events and prolongs survival [331]. Other strategies that have been considered for inhibiting metastasis and premetastatic niches include inhibiting CXCR4 and preventing stiffening of the ECM by targeting *LOX* and *LOXL2* enzymes [332]. Concurrent with this, ongoing improvements in disease monitoring and therapy delivery are also changing the paradigm of metastatic cancer treatment. However, many challenges remain regarding the sensitivity of standard imaging tools for detecting dormant cancer stages below their thresholds. A liquid biopsy is one technique to assess the metastatic process in a relatively painless way by measuring changes in the amount and dynamics of circulating tumor DNA, which can often anticipate changes visible on scans [333]. However, during dormant stages, tumor cells are likely to release very little DNA. To mitigate these limitations, new approaches leverage epigenetic and structural characteristics of circulating tumor DNA (ctDNA), including methylation patterns and fragmentomic parameters such as fragment length distributions, termination patterns, and inferred nucleosome positioning. Such new therapies hold promise for better identification of minimal disease or dormancy states but still require rigorous prospective studies and standardization before their use in clinical settings. New complementary single-cell and rare-cell analytics, such as circulating tumor cell (CTC) analysis and high-dimensional immune cell phenotyping, provide enhanced biological insights concerning dormancy and immune evasion. Nevertheless, such factors relating to scalability, cost, and inter-platform validity currently represent formidable challenges to their use in clinical settings for minimal residual disease or metastatic dormancy. Parallel advances in therapeutic delivery also promote chronic disease management. Nanomedicine platforms have been shown to increase drug accumulation in metastatic lesions [334], while depot systems formulated from biomaterials, such as injectable immunostimulatory hydrogels, enable local immune stimulation to prevent recurrence [335]. Breakthroughs in cellular engineering, using CRISPR-edited T cells [336], tumor-targeting MSC therapies [337], and universal iPSC NK/T cells products [338], now offer modular and ready-to-use approaches to systemic immunotherapy. Taken together, all of these advances demonstrate a shift from indiscriminate, purely cytotoxic cancer therapies toward fully interdisciplinary, precision-oriented approaches to cancer management. Using this paradigm shift approach against cancer metastasis, metastatic cancer can be practically and fully controlled and managed.

## Figures and Tables

**Table 1:** EMT, plasticity, and invasion initiation.

Gene Name	Function	Description	Ref.
*SNAIL*	EMT, CAF activation, Prostaglandin E2 (PGE2)	Induces EMT and invasion in carcinoma cells (Represses E-cadherin). Required for CAF activation; CAFs secrete PGE_2_ and cytokines to drive tumor invasion (Regulates mesenchymal differentiation, wound healing).	[19]
*TWIST1*	EMT, PDGFRα, Src, invadopodia	*TWIST1* EMT TF: invadopodia formation (via PDGFRα/Src) Drives EMT, motility, invadopodia-mediated ECM degradation (Not directly studied). Upregulated in CAFs of many tumors: Twist1 promotes invasion and tumor growth.	[20]
*SP1*	Pan-cancer TF, survival/invasion, WNT/β-catenin	Pan-cancer TF: induces WNT signaling, survival, and invasion. Master regulator of metastasis genes; enhances WNT/β-catenin signaling in tumor cells. Drives expression of angiogenic factors (e.g., VEGF); WNT signals from stroma to endothelium.	[21,22]
*EZH2*	H3K27me3 silencing, EMT, stromal remodeling	*EZH2* Histone methyltransferase: epigenetic silencer of adhesion genes. Silences E-cadherin/epithelial genes, activating EMT and invasion (May promote EndMT by methylating endothelial promoters). Drives fibroblast-to-myofibroblast transition; promotes fibrotic stroma. Regulates mesenchymal stem cell (MSC) proliferation/differentiation (Wound healing analogies).	[23,24,25]
*YBX1*	EMT, stress survival, drug resistance	*YBX1* RNA/DNA-binding protein; induces EMT and stress survival. Activates EMT-related mRNAs (*Snail*, *Twist*) and drug resistance pathways in tumors. Regulates VEGF expression under hypoxia, promoting angiogenesis. Contributes to fibroblast activation by stabilizing cytokine mRNAs. Modulates MSC plasticity and response to microenvironmental stress.	[26,27]
*ZEB2*	EMT TF; metastasis and stromal/EndMT links	*ZEB2* EMT transcription factor; represses epithelial genes. Drives EMT and mesenchymal phenotype in carcinoma cells (analogous to *Snail*/*Zeb1*) (Possible role in EndMT/transdifferentiation of endothelium). Induces fibroblast-like program in epithelial and endothelial cells. In MSCs, *ZEB2* may regulate multilineage differentiation toward mesenchyme.	[28,29]

**Table 2:** Tumor-stroma interaction, inflammation, and ECM remodeling.

Gene Name	Function	Description	Ref.
*IL-6*	STAT3/EMT; CAF source; angiogenesis	Pro-inflammatory cytokine; activates *JAK*/*STAT3*, EMT receptor-expressing carcinoma cells undergo *STAT3*-dependent EMT and proliferation. Promotes angiogenesis and leukocyte recruitment in tumor vessels. Secreted by CAFs (and tumor cells); drives EMT/migration of cancer cells. IL-6 can recruit and modulate MSCs (MSC chemotaxis, differentiation)	[42,43]
*CXCL8* (IL-8)	Angiogenesis, EMT/invasion, CXCR1/2	Promotes angiogenesis, EMT, and invasion. Tumor-derived IL-8 induces autocrine EMT/invasion and survival. Potent angiogenic factor; stimulates endothelial proliferation and vessel permeability. CAFs secrete IL-8 to boost tumor angiogenesis and invasion. MSCs respond to IL-8 (via CXCR1/2), promotes MSC migration and possibly MSC-to-CAF transition.	[44,45]
*CXCL1*	Neutrophil recruitment, angiogenesis	CXCL1 Chemokine (ELR^+^); recruits neutrophils, fosters angiogenesis. Tumor-secreted CXCL1 creates a pro-inflammatory niche for invasion (by analogy to IL-8). Angiogenic; contributes to neovascularization (via CXCR2). Expressed by CAFs and TAMs; enhances tumor cell motility and chemoresistance (paracrine). May attract MSCs to the tumor; role is less defined than IL-8	[46,47]
*MMP9*	ECM degradation, growth-factor activation, angiogenesis	MMP9 Secreted matrix metalloprotease; cleaves ECM, activates growth factors. Tumor cells secrete MMP9 to breach the basement membrane (promoting intravasation). Degrades endothelial basement membranes to enable angiogenesis and metastasis. CAFs/myofibroblasts produce MMP9 to remodel the stroma and release pro-metastatic signals. MSCs secrete MMP9 to facilitate migration; MSC-derived MMPs shape the metastatic niche.	[48,49]
*MMP1*	Interstitial collagenase, invasion/angiogenesis	MMP1 Interstitial collagenase; degrades type-I/III collagen. Tumor-derived MMP1 promotes invasion through dense stroma, enabling new vessel growth by remodeling perivascular ECM. CAFs produce MMP1 to stiffen or remodel the matrix, enabling tumor spreading. MSCs may also express MMP1 in differentiation contexts.	[50,51]

## Data Availability

No new data were created or analyzed in this study. Data sharing is not applicable to this article.
